# Association of KCNB1 polymorphisms with lipid metabolisms and insulin resistance: a case-control design of population-based cross-sectional study in Chinese Han population

**DOI:** 10.1186/s12944-015-0115-1

**Published:** 2015-09-17

**Authors:** Yuncui Yu, Jing Wang, Ruiying Kang, Jing Dong, Yuxiang Zhang, Fen Liu, Yuxiang Yan, Rong Zhu, Lili Xia, Xiaoxia Peng, Ling Zhang, Dian He, Gaisano Herbert, Zhenwen Chen, Yan He

**Affiliations:** School of Public Health, Capital Medical University, No.10 Xitoutiao, Youanmen, Fengtai District, Beijing, 100069 PR China; Departments of Emergency, Beijing Xuanwu Hospital, Capital Medical University, No.45Changchun Street, Xuanwu District, Beijing, 100053 Beijing, PR China; Departments of Medicine and Physiology, University of Toronto, 315 Bloor Street West, Toronto, Ontario Canada; School of Basic Medicine, Capital Medical University, No.10 Xitoutiao, Youanmen, Fengtai District, Beijing, 100069 PR China

**Keywords:** KCNB1 polymorphisms, Kv2.1 channel, Lipid metabolism, Insulin resistance

## Abstract

**Background:**

In our previous study, we had assessed in the Chinese Han population the association of KCNB1 rs1051295 with metabolic traits indicating metabolic syndrome, and showed that KCNB1 rs1051295 genotype TT was associated with increase of waist to hip ratio (WHR), fasting insulin (FINS), triglycerides (TG) and decreased insulin sensitivity at basal condition. Here, we aimed at detecting whether there were associations between other tag SNPs of KCNB1 and favorable or unfavorable metabolic traits.

**Methods:**

We conducted a case–control design of population-based cross-sectional study to investigate the association between each of the 22 candidates tag SNPs of KCNB1 and metabolic traits in a population of 733 Chinese Han individuals. The association was assessed by multiple linear regression analysis or unconditional logistic regression analysis.

**Results:**

We found that among the 22 selected tag SNPs, four were associated with an increase (rs3331, rs16994565) or decrease (rs237460, rs802950) in serum cholesterol levels; two of these (rs237460, rs802590) further associated or were associated with reduced serum LDL-cholesterol. Two novel tag SNPs (rs926672, rs1051295) were associated with increased serum TG levels. We also showed that KCNB1 rs926672 associated with insulin resistance by a case–control study, and two tag SNPs (rs2057077and rs4810952) of KCNB1 were associated with the measure of insulin resistance (HOMA-IR) in a cross-section study.

**Conclusion:**

These results indicate that KCNB1 is likely associated with metabolic traits that may either predispose or protect from progression to metabolic syndrome. This study provides initial evidence that the gene variants of KCNB1, encoding Kv2.1 channel, is associated with perturbation of lipid metabolism and insulin resistance in Chinese Han population.

## Background

KCNB1, also known as Kv2.1, is located on chromosome 20q13.2, and is mainly expressed in the brain cortex and hippocampus, islet β-cells, cardiac atrium and ventricle, skeletal muscles and some other tissues [1]. Our recently reported case–control study in Chinese Han population showed a strong association of KCNB1 rs1051295 genotype TT with an increased risk of becoming afflicted with metabolic syndrome leading to T2D. Specifically, we showed the genotype TT of this SNP was associated with several metabolic traits of metabolic syndrome, including increased waist to hip ratio (WHR), fasting insulin (FINS) levels, increased serum triglyceride (TG) levels, and decreased insulin sensitivity at basal condition [2].

Kv2.1 channel’s role in secretion is attributed not only to the regulation of membrane excitability but also to a more direct role in the exoctyotic machinery. Up-regulation of Kv2.1 channel in PC12 [3], bovine chromaffin cells [4], rat dorsal root ganglion cells [5] and rodent and human islet β-cells, [6] facilitated exocytotic SNARE (soluble N-ethylmaleimide-sensitive factor attachment protein receptor) complex assembly initiated by direct interaction of its C-terminus with SNARE protein syntaxin-1A and subsequent further assembly with VAMP2 and SNAP25. In β-cells, these Kv2.1-SNARE complex interactions can enhance insulin secretion [6]. To our knowledge, there have been no in vivo or in vitro studies to demonstrate a possible role of Kv2.1 channel in metabolism to account for the perturbed metabolic traits we had reported [2]. However, in support of this possibility that Kv channels could be involved in body metabolic functions, Chandy and colleagues recently demonstrated that ShK-186, a selective and potent blocker of voltage-gated Kv1.3 channel, reduced weight gain, adiposity, and fatty liver with resultant enhancement of peripheral insulin sensitivity; and also decreased serum levels of cholesterol, glucose, HbA1c, insulin, and leptin [7].

To delve deeper into the role of KCNB1-encoded Kv2.1 in metabolic syndrome, we examined the associations between the tag SNPs of KCNB1 and different metabolic traits of metabolic syndrome. This study explores the possible role of Kv2.1 and its influence in the body’s metabolism leading to T2D.

## Results

### General information of the participants

Of the 733 participants, mean age was 39.9 ± 10.4 years old, with males constituting 43 % (315/733) of the total participants. FPG, TC, TG, HDL-C and LDL-C for the participants was 4.9 ± 0.5, 4.6 ± 0.9, 1.1 ± 0.6, 1.7 ± 0.3 and 2.7 ± 0.7 mmol/L, respectively, and the HOMA-IR and HOMA-B% was 1.2 ± 0.4and 108.7 ± 31.9 %, respectively.

### SNPs of KCNB1 associated with changes in serum TC or LDL-C

rs237460: Compared with genotype AA, genotype GG (*P* = 0.02, b = -0.11 (-0.19,-0.02)) and GA (*P* = 0.02, b = -0.19(-0.34, -0.04)) was associated with decreased serum TC levels. We also found that serum levels of LDL-C of genotype GA (2.60 mmol/L) (*P* = 0.005, b = -0.18(-0.31, -0.06)) and GG (2.64 mmol/L) (*P* = 0.07, b = -0.07 (-0.14, 0.004)) was lower or trend to be lower than that in genotype AA (2.78 mmol/L) (Table 1).

**Table 1:** The tag SNPs of KCNB1 associated with lipid metabolism and insulin resistance

	Genotype	M/F	Age	BMI	WHR	TC	TG	HDL-C	LDL-C	FPG	FINS	HOMA-IR	HOMA-B
rs16994565	TT	267/341	39.68 ± 10.05	23.10 ± 2.48	0.80 ± 0.07	4.60 ± 0.84	1.11 ± 0.61	1.67 ± 0.34	2.67 ± 0.71	4.94 ± 0.48	8.91 ± 3.29	1.15 ± 0.42	108.58 ± 32.51
	TC	40/66	39.91 ± 11.26	22.95 ± 2.73	079 ± 0.07	4.46 ± 0.80	1.02 ± 0.57	1.66 ± 0.30	2.54 ± 0.67	4.90 ± 0.47	8.88 ± 3.26	1.14 ± 0.42	109.46 ± 30.33
	CC	3/4	36.00 ± 9.52	22.39 ± 2.90	0.77 ± 0.07	3.75 ± 1.71	0.98 ± 0.31	1.64 ± 0.25	2.49 ± 0.34	4.85 ± 0.33	8.63 ± 1.09	1.10 ± 0.15	110.49 ± 19.00
*P* ^*1*^		0.5	0.62	0.67	0.51	0.01	0.31	0.95	0.18	0.75	0.97	0.95	0.82
*P* ^*2*^(TC vs CC)				0.83,0.21(-1.71,2.13)	0.23,0.02(-0.01,0.06)	0.08,0.58(-0.06,1.23)	0.99,0.001(-0.39,0.39)	0.90,-0.01(-0.22,0.20)	0.95,-0.01(-0.50,0,48)	0.84,-0.03(-0.35,0.29)	0.81,0.31(-2.17,2.79)	0.78,-0.05(-0.28,0.37)	0.80,2.81(-19.05,24.67)
*P* ^2^(TT vs CC)				0.60,0.23(-0.62,1.08)	0.24,0.01(-0.01,0.03)	0.02,0.36(0.06,0.66)	0.74,0.03(-0.17,0.24)	0.93,0.01(-0.11,0.12)	0.73,0.04(-0.20,0.29)	0.91,0.01(-0.16,0.17)	0.78,0.18(-1.04,1.39)	0.73,0.03(-0.13,0.18)	0.92,0.64(-11.04,12.31)
rs237460	GG	101/128	39.34 ± 10.39	22.92 ± 2.58	0.795 ± 0.07	4.51 ± 0.91	1.07 ± 0.56	1.66 ± 0.30	2.64 ± 0.73	4.91 ± 0.47	8.63 ± 3.26	1.11 ± 0.42	106.55 ± 29.15
	GA	157/204	39.85 ± 10.20	23.13 ± 2.52	0.798 ± 0.07	4.55 ± 0.82	1.11 ± 0.60	1.65 ± 0.34	2.60 ± 0.69	4.94 ± 0.48	9.00 ± 3.25	1.16 ± 0.42	109.16 ± 32.36
	AA	53/80	40.09 ± 10.36	23.20 ± 2.45	0.796 ± 0.07	4.73 ± 0.80	1.12 ± 0.64	1.72 ± 0.34	2.78 ± 0.69	4.94 ± 0.46	9.11 ± 3.34	1.17 ± 0.42	110.98 ± 35.76
*P* ^*1*^		0.71	0.77	0.52	0.87	0.04	0.67	0.13	0.06	0.82	0.29	0.33	0.24
*P* ^2^(GA vs AA)				0.66,-0.10(-0.56,0.36)	0.89,-0.001(-0.01,0.01)	0.02,-0.19(-0.34,-0.04)	0.62,-0.03(-0.14,0.08)	0.07,-0.06(-0.12,0.01)	0.005,-0.18(-0.31,-0.06)	0.98,-0.001(-0.09,0.09)	0.66,-0.15(-0.79.0.50)	0.75,-0.01(-0.10,0.07)	0.49,-2.23(-8.63,4.17)
*P* ^2^(GG vs AA)				0.26,-0.14(-0.39,0.11)	0.51,-0.002(-0.01,0.003)	0.02,-0.11(-0.19,-0.02)	0.31,-0.03(-0.09,0.03)	0.15,-0.02(-0.06,0.10)	0.07,-0.07(-0.14,0.004)	0.73,-0.01(-0.06,0.04)	0.15,-0.26(-0.61,0.10)	0.19-,0.03(-0.08,0.02)	0.13,-2.53(-5.82,0.77)
rs3331	TT	270/348	39.48 ± 10.06	23.08 ± 2.51	0.80 ± 0.07	4.59 ± 0.84	1.11 ± 0.60	1.67 ± 0.34	2.66 ± 0.71	4.93 ± 0.48	8.90 ± 3.28	1.15 ± 0.42	108.53 ± 32.33
	TC	40/59	41.15 ± 11.36	23.08 ± 2.61	0.80 ± 0.06	4.48 ± 0.79	1.03 ± 0.59	1.63 ± 0.27	2.57 ± 0.68	4.91 ± 0.46	8.97 ± 3.29	1.15 ± 0.43	110.04 ± 30.98
	CC	1/4	39.2 ± 11.48	22.64 ± 3.22	0.76 ± 0.06	3.57 ± 2.05	0.94 ± 0.33	1.69 ± 0.15	2.58 ± 0.38	4.85 ± 0.25	7.94 ± 1.16	1.02 ± 0.16	104.26 ± 17.80
*P* ^*1*^		0.48	0.32	0.93	0.31	0.02	0.42	0.71	0.47	0.8	0.79	0.78	0.97
*P* ^2^(TC vs CC)				0.96,0.06(-2.11,2.24)	0.22,0.03(-0.02,0.07)	0.03,0.83(0.09,1.57)	0.94,-0.02(-0.50,0.47)	0.83,-0.03(-0.25,0.20)	0.73,-0.10(-0.68,0.48)	0.82,0.04(-0.33,0.42)	0.55,0.90(-2.07,3.87)	0.54,0.12(-0.27,0.51)	0.69,5.43(21.04,31.89)
*P* ^2^ (TT vs CC)				0.95,0.03(-0.99,1.05)	0.43,0.01(-0.01,0.03)	0.01,0.49(0.13,0.84)	0.79,0.03(-0.21,0.27)	0.81,0.02(-0.12,0.16)	0.97,-0.01(-0.29,0.28)	0.80,0.03(-0.17,0.22)	0.58,0.40(-1.03,1.84)	0.57,0.05(-0.13,0.24)	0.77,2.08(-11.64,15.80)
rs802950	CC	212/276	39.63 ± 10.27	23.09 ± 2.52	0.80 ± 0.07	4.60 ± 0.86	1.12 ± 0.60	1.67 ± 0.33	2.67 ± 0.71	4.92 ± 0.48	8.85 ± 3.29	1.14 ± 0.42	108.67 ± 31.87
	AC	89/118	39.58 ± 10.25	23.06 ± 2.52	0.79 ± 0.07	4.47 ± 0.84	1.05 ± 0.58	1.64 ± 0.33	2.58 ± 0.69	4.96 ± 0.49	9.03 ± 3.21	1.17 ± 0.41	108.84 ± 32.97
	AA	10/19	42.45 ± 10.06	22.96 ± 2.61	0.80 ± 0.06	4.90 ± 0.85	1.12 ± 0.73	1.79 ± 029	2.79 ± 0.68	4.94 ± 0.35	9.02 ± 3.39	1.16 ± 0.44	108.07 ± 29.56
*P* ^1^		0.64	0.35	0.96	0.29	0.02	0.44	0.07	0.18	0.58	0.79	0.75	0.58
*P* ^2^(AC vs AA)				0.69,0.18(-0.71,1.07)	0.32,-0.01(-0.03,0.01)	0.03,-0.36(-0.68,-0.05)	0.56,-0.06(-0.28,0.15)	0.06,-0.12(-0.24,0.01)	0.14,-0.19(-0.45,0.06)	0.36,0.08(-0.09,0.24)	0.98,-0.02(-1.29,1.25)	0.93,0.01(-0.16,0.17)	0.78,-1.77(-14.19,10.66)
*P* ^2^(CC vs AA)				0.65,0.10(-0.34,0.54)	0.88,0.001(-0.01.0.01)	0.17,-0.11(-0.26,0.05)	0.99,0.001(-0.11,0.11)	0.12,-0.05(-0.11,0.01)	0.61,-0.03(-0.16,0.09)	0.85,0.01(-0.08,0.09)	0.61,-0.16(-0.77,0.46)	0.66,-0.02(-0.10,0.06)	0.72,-1.06(-6.79,4.67)
rs926672	AA	97/145	39.66 ± 10.39	22.91 ± 2.43	0.80 ± 0.07	4.57 ± 0.90	1.10 ± 0.58	1.69 ± 0.35	2.63 ± 0.74	4.93 ± 0.44	8.66 ± 3.15	1.12 ± 0.41	106.19 ± 29.59
	AT	165/190	39.69 ± 10.29	23.28 ± 2.61	0.80 ± 0.07	4.61 ± 0.81	1.15 ± 0.65	1.65 ± 0.32	2.68 ± 0.69	4.94 ± 0.51	9.23 ± 3.41	1.19 ± 0.44	111.47 ± 34.21
	TT	48/76	39.89 ± 10.00	23.78 ± 2.42	0.79 ± 0.07	4.44 ± 0.86	0.94 ± 0.45	1.66 ± 0.31	2.61 ± 0.65	4.91 ± 0.43	8.43 ± 3.01	1.08 ± 0.39	105.49 ± 29.76
*P* ^1^		0.17	0.98	0.08	0.21	0.19	0.004	0.44	0.53	0.83	0.02	0.02	0.07
*P* ^2^(AT vs TT)				0.13,0.38(-0.11,0.88)	0.17,0.01(-0.003,0.02)	0.049,0.16(0.00,0.32)	0.002,0.19(0.07,0.30)	0.90,0.004(-0.06,0.07)	0.46,0.05(-0.08,0.18)	0.62,0.02(-0.07,0.12)	0.03,0.74(0.06,1,42)	0.02,0.10(0.01,0.19)	0.09,5.66(-0.86,12.18)
*P* ^2^(AA vs TT)				0.60,0.07(-0.18,0.31)	0.15,0.004(-0.001,0.01)	0.16,0.06(-0.13,0.16)	0.004,0.08(0.02,0.13)	0.46,0.01(-0.02,0.05)	0.75,0.01(-0.06,0.08)	0.62,0.01(-0.03,0.06)	0.53,0.11(-0.23,0.44)	0.45,0.02(-0.03,0.06)	0.88,0.24(-2.83,3.31)
rs1051295	TT	111/147	39.96 ± 10.82	22.97 ± 2.50	0.80 ± 0.07	4.65 ± 0.92	1.13 ± 0.58	1.67 ± 0.35	2.71 ± 0.80	4.93 ± 0.51	9.08 ± 3.34	1.17 ± 0.43	110.43 ± 33.11
	TC	153/203	40.33 ± 10.16	23.10 ± 2.50	0.79 ± 0.07	4.54 ± 0.83	1.12 ± 0.65	1.66 ± 0.34	2.63 ± 0.64	4.94 ± 0.44	8.95 ± 3.26	1.15 ± 0.42	108.35 ± 31.32
	CC	51/68	38.3 ± 9.81	23.29 ± 2.66	0.79 ± 0.07	4.49 ± 0.73	0.99 ± 0.45	1.68 ± 0.28	2.61 ± 0.67	4.93 ± 0.50	8.52 ± 3.06	1.10 ± 0.39	105.98 ± 31.02
*P* ^1^		1	0.18	0.51	0.42	0.13	0.08	0.76	0.22	0.9	0.29	0.24	0.44
*P* ^2^(TC vs CC)				0.16,-0.35(-0.83,0.13)	0.42,-0.004(-0.01,001)	0.88,0.11(-0.15,0.18)	0.10,0.10(-002,0.22)	0.51,-0.02(-0.09,0.04)	0.78,-0.02(-0.14,0.11)	0.73,-0.02(-0.11,0.07)	0.16,0.48(-0.19,1.15)	0.16,0.06(-0.03,0.15)	0.21,4.07(-2.23,10.36)
*P* ^2^(TT vs CC)				0.09,-0.22(-0.48,0.04)	0.60,0.001(-0.004,0.01)	0.19,0.06(-0.03,0.15)	0.03,0.06(0.01,0.12)	0.66,-0.01(-0.04,0.03)	0.39,0.03(-0.04,0.11)	0.49,-0.02(-0.07,0.03)	0.11,2.92(-0.06,0.65)	0.09,0.04(-0.01,0.09)	0.09,2.96(-0.45,6.36)
rs2057077	CC	102/160	39.42 ± 10.30	22.89 ± 2.52	0.79 ± 0.07	4.55 ± 0.92	1.07 ± 0.58	1.67 ± 0.33	2.62 ± 0.72	4.93 ± 0.45	8.64 ± 3.18	1.11 ± 0.41	105.91 ± 28.55
	TC	170/193	39.74 ± 10.28	23.22 ± 2.58	0.80 ± 0.07	4.58 ± 0.82	1.13 ± 0.61	1.65 ± 0.33	2.68 ± 0.69	4.94 ± 0.48	9.26 ± 3.36	1.19 ± 0.43	111.55 ± 34.05
	TT	40/60	40.71 ± 10.32	23.04 ± 2.27	0.79 ± 0.07	4.59 ± 0.80	1.04 ± 0.60	1.67 ± 0.33	2.66 ± 0.72	4.90 ± 0.51	8.29 ± 3.03	1.06 ± 0.39	105.53 ± 32.51
*P* ^1^		0.12	0.57	0.26	0.52	0.91	0.25	0.73	0.59	0.76	0.01	0.005	0.02
*P* ^2^(TC vs TT)				0.60,0.14(-0.39,0.67)	0.86,0.001(-0.01,0.01)	0.88,0.01(-0.16,0.18)	0.19,0.08(-0.04,0.21)	0.96,0.002(-0.07,0.07)	0.83,0.03(-0.13,0.16)	0.36,0.05(-0.05,0.15)	0.01,0.92(0.19,1.65)	0.008,0.13(0.03,0.23)	0.17,5.06(-2.17,12.29)
*P* ^2^(CC vs TT)				0.83,-0.03(-0.29,0.24)	0.51,0.002(-0.004,0.01)	0.90,0.006(-0.09,0.10)	0.31,0.03(-0.03,0.09)	0.95,0.001(-0.03,0.04)	0.95,-0.003(-0.08,0.07)	0.27,0.03(-0.02,0.08)	0.36,0.17(-0.19,0.53)	0.29,0.03(-0.02,0.72)	0.83,-0.35(-3.63,2.92)
rs4810952	TT	81/126	39.43 ± 10.15	22.97 ± 2.46	0.79 ± 0.07	4.58 ± 0.89	1.08 ± 0.58	1.68 ± 0.34	2.65 ± 0.73	4.94 ± 0.43	8.40 ± 3.18	1.10 ± 0.41	103.09 ± 28.72
	TC	170/194	39.82 ± 10.33	23.16 ± 2.61	0.80 ± 0.07	4.59 ± 0.82	1.14 ± 0.62	1.66 ± 0.33	2.66 ± 0.71	4.93 ± 0.50	9.30 ± 3.37	1.20 ± 0.43	112.54 ± 34.05
	CC	60/91	39,79 ± 10.27	23.02 ± 2.40	0.79 ± 0.06	4.59 ± 0.87	1.02 ± 0.57	1.65 ± 0.31	2.61 ± 0.64	4.92 ± 0.46	8.62 ± 3.04	1.11 ± 0.39	107.17 ± 30.30
*P* ^1^		0.14	0.9	0.67	0.25	0.56	0.11	0.56	0.76	0.88	0.004	0.004	0.01
*P* ^2^(TC vs TT)				0.91,0.03(-0.38,0.43)	0.87,-0.001(-0.01,0.01)	0.85,-0.01(-0.15,0.13)	0.61,0.03(-0.07,0.12)	0.78,-0.01(-0.06,0.05)	0.74,-0.02(-0.14,0.10)	0.41,-0.03(-0.11,0.04)	0.003,0.86(0.30,1.43)	0.004,0.11(0.04,0.18)	<0.001,9.60(4.27,14.93)
*P* ^2^(CC vs TT)				0.93,0.01(-0.23,0.25)	0.52,-0.002(-0.01,0.003)	0.30,-0.05(-0.13,0.04)	0.26,-0.03(-0.09,0.02)	0.30,-0.02(-0.06,0.02)	0.52,-0.02(-0.09,0.05)	0.50,-0.02(-0.06,0.03)	0.48,0.12(-0.21,0.44)	0.59,0.01(-0.03,0.06)	0.15,2.18(-0.79,5.16)

Data are present as mean ± SD, *M/F* male/female, *WHR* Waist to hip ratio, *TG* triglycerides, *TC* serum total cholesterol, *HDL-C* high-density lipoprotein cholesterol, *LDL-C* low-density lipoprotein cholesterol, *FPG* fasting plasma glucose, *FINS* fasting serum insulin, *P* ^*1*^ from one-way ANOVA test except *P* ^1^for M/F from *χ* ^2^ test, *P* ^2^ from multiple linear regression analysis with b and 95 % CIs (adjusted for age and gender)

rs802590: Genotype AC was associated with decreased serum levels of TC (*P =* 0.03, b = -0.36(-0.68, -0.05)) and decreased serum levels of LDL-C (*P* = 0.06, b = -0.12 (-0.24, 0.01)) compared with genotype CC, whereas genotype AA showed no association (for TC: *P* = 0.17, b = -0.11(-0.26, 0.05); for LDL-C:*P* = 0.61, b = -0.03(-0.16,0.09)) (Table 1).

rs16994565: Compared with genotype CC, the serum TC levels in genotype TT and TC increased by 22.7 % (*P* = 0.02, b = 0.36(-0.06, 0.66)) and 18.9 % (*P* = 0.08, b = 0.58 (-0.06, 1.23)), respectively (Table 1).

rs3331: genotype TT (*P* = 0.01, b = 0.49 (0.13, 0.84) and TC (*P =* 0.03, b = 0.83 (0.09, 1.57)) was associated with increased serum levels of TC compared to genotype CC. Although the TG in genotype TT (1.11 mmol/L) increased 15.4 % than genotype CC (0.94 mmol/L), it did not reach a significant level (*P* = 0.79, b = 0.03(-0.21, 0.27)) (Table 1).

### SNPs of KCNB1 associated with changes in serum TG

rs926672: Compared with genotype TT, the serum TG levels in genotype AA (*P* = 0.004, b = 0.08(0.02, 0.13)) and AT (*P* = 0.002, b = 0.19(0.07, 0.30)) increased 14.5 and 18.3 %, respectively. In addition, genotype AT was associated with increased TC levels (*P* = 0.049, b = 0.16(0.00, 0.32)), insulin resistance (*P* = 0.03, b = 0.74(0.06, 1.42)), and fasting insulin (FINS) (*P* = 0.02, b = 0.10 (0.01, 0.19)) compared with genotype TT, and showed a trend in increasing islet β-cell function by HOMA-B% (*P* = 0.09, b = 5.66(-0.86, 12.18)) (Table 1).

rs1051295: Compared with genotype CC, both of genotype TT (*P* = 0.03, b = 0.06 (0.01, 0.12)) and TC (*P* = 0.10, b = 0.10(-002, 0.22)) was associated or inclined to be associated with increased serum TG levels, in line with our previous report [2]. We also found that genotype TT tended to be associated with increased BMI (*P* = 0.09, b = -0.22 (-0.48, 0.04)), HOMA-IR (*P* = 0.09, b = 0.04(-0.01, 0.09)), FINS (*P* = 0.11, b = 2.92(-0.06, 0.65)) and HOMA-B (*P* = 0.09, b = 5.66(-0.86, 12.18)) compared with genotype CC (Table 1).

### SNPs of KCNB1 associated with the measure of insulin resistance by HOMA-IR

rs4810952: Compared with genotype TT, genotype TC was associated with increased HOMA-IR (*P* = 0.01, b = -9.84(-16.65,-3.02)), FINS (*P* = 0.003, b = 0.86(0.30, 1.43)) and the HOMA-B% (*P* < 0.001, b = 9.60(4.27, 14.93)) (Table 1).

rs2057077: Compared with genotype TT (1.06 mmol/L and 8.29mUI/L), HOMA-IR and FINS of TC (1.19 mmol/L and 9.26mUI/L) increased 12.3 % (*P* = 0.008, b = 0.13(0.03, 0.23)) and 11.70 % (*P* = 0.01, b = 0.92 (0.19, 1.65)), respectively (Table 1).

### SNPs of KCNB1 associated with insulin resistance (IR)

According to the definition of insulin resistance, we identified 199 IR subjects and 534 Non-IR subjects from 733 participants, and the prevalence of IR were 27.15 % (199/733) in this study. The clinical anthropometric and biochemical characteristics of the participants are summarized in Table 2. Among 22 available tag SNPs of KCNB1, the rs926672 (*P* = 0.03) and rs2057077 (*P* = 0.04) were associated with insulin resistance by univariate analysis. However, only rs926672 was associated with IR (*P* = 0.03) after being adjusted for age and gender (Table 3). We found that the risk for the carrier of genotype AT suffering from insulin resistance was 1.77 fold to that of genotype TT (*P* = 0.03, OR = 1.77, (1.07-2.93)). In the dominant or recessive model, neither allele T (genotype TT and AT) (*P* = 0.50, OR = 1.13(0.79, 1.62)) nor allele A (genotype AA and AT) (*P* = 0.057, OR = 1.60(0.99-2.60)) was associated with IR. Although we had already found that KCNB1 rs1051295 genotype TT was associated with T2D and decreased insulin sensitivity in our previous study, no association was observed for rs1051295 and insulin resistance in either dominant model (*P* = 0.72, OR = 1.07 (0.75,1.51)) or recessive model (*P* = 0.24, OR = 0.75(0.47,1.20)) by univariate or multivariate analysis in this study.

**Table 2:** Distribution of clinical and anthropometric characteristics in IR and Non-IR subjects

Variables	IR (*n* =199)	Non-IR (n = 534)	*P*
Age (years)	39(31-48)	40(31-48)	0.87
M/F	111/88	315/418	<0.001
BMI (kg/m^2^)	24.55 ± 2.20	22.54 ± 2.42	<0.001
WHR	0.82 ± 0.07	0.79 ± 0.07	<0.001
TG (mmol/L)	1.17(0.83-1.67)	0.85(0.67-1.21)	<0.001
TC (mmol/L)	4.63 ± 0.85	4.55 ± 0.85	0.27
HDL-C(mmol/L)	1.57 ± 0.34	1.70 ± 0.32	<0.001
LDL-C(mmol/L)	2.70 ± 0.72	2.64 ± 0.70	0.29
FPG (mmol/L)	5.06 ± 0.51	4.89 ± 0.45	<0.001
FINS(mU/L)	12.98 ± 2.23	7.41 ± 2.08	<0.001

Data are present as mean ± SD, or median (25%quatile-75%quatile). *M/F* male/female, *W/H ratio* waist/hip circumference, *TG* triglycerides, *TC* serum total cholesterol, *HDL-C* high-density lipoprotein cholesterol, *LDL-C* low-density lipoprotein cholesterol, *FPG* fasting plasma glucose, *P* from independent-sample *t* test except *P* for M/F from *χ* ^2^ test

**Table 3:** Distribution of the tag SNPs of KCNB1in case–control study

SNP ID	IR(*n* = 199)	Non-IR(*n* = 534)	*χ* ^2^	*P* ^*1*^	*P* ^*2*^	OR (95 % CI)
rs926672			6.76	0.03		
AA	60	182				
AT	109	246				
TT	24	100				
AA vs TT					0.25	1.37(0.80-2.35)
AT vs TT					0.03	1.77(1.07-2.93)
AA vs AT					0.18	1.29(0.89-1.88)
Dominent model				0.40	0.50	1.13(0.79-1.62)
Recessive model				0.04	0.06	1.60(0.99-2.60)
rs2057077			6.33	0.04		
CC	62	200				
TC	111	252				
TT	20	80				
CC vs TT					0.44	0.89(0.67-1.19)
CC vs TC					0.11	1.35(0.94-1.95)
TT vs TC					0.06	0.59(0.34-1.01)
Dominent model				0.18	0.27	1.22(0.86-1.74)
Recessive model				0.11	0.13	0.66(0.39-1.12)

*P* ^*1*^ from *χ* ^2^ test, *P* ^*2*^ from Logistic regression analysis and adjusted for age, gender, Dominant Model TT + AT compared with AA, *Recessive Model* AA + AT compared with TT for rs926672, *Dominant Model* TT + TC compared with CC, *Recessive Model* CC + TC compared with TT for rs2057077, *OR* odds ratio, *CI* confidence interval

### Haplotypes Analysis of KCNB1 with Insulin Resistance

**Fig. 1:**
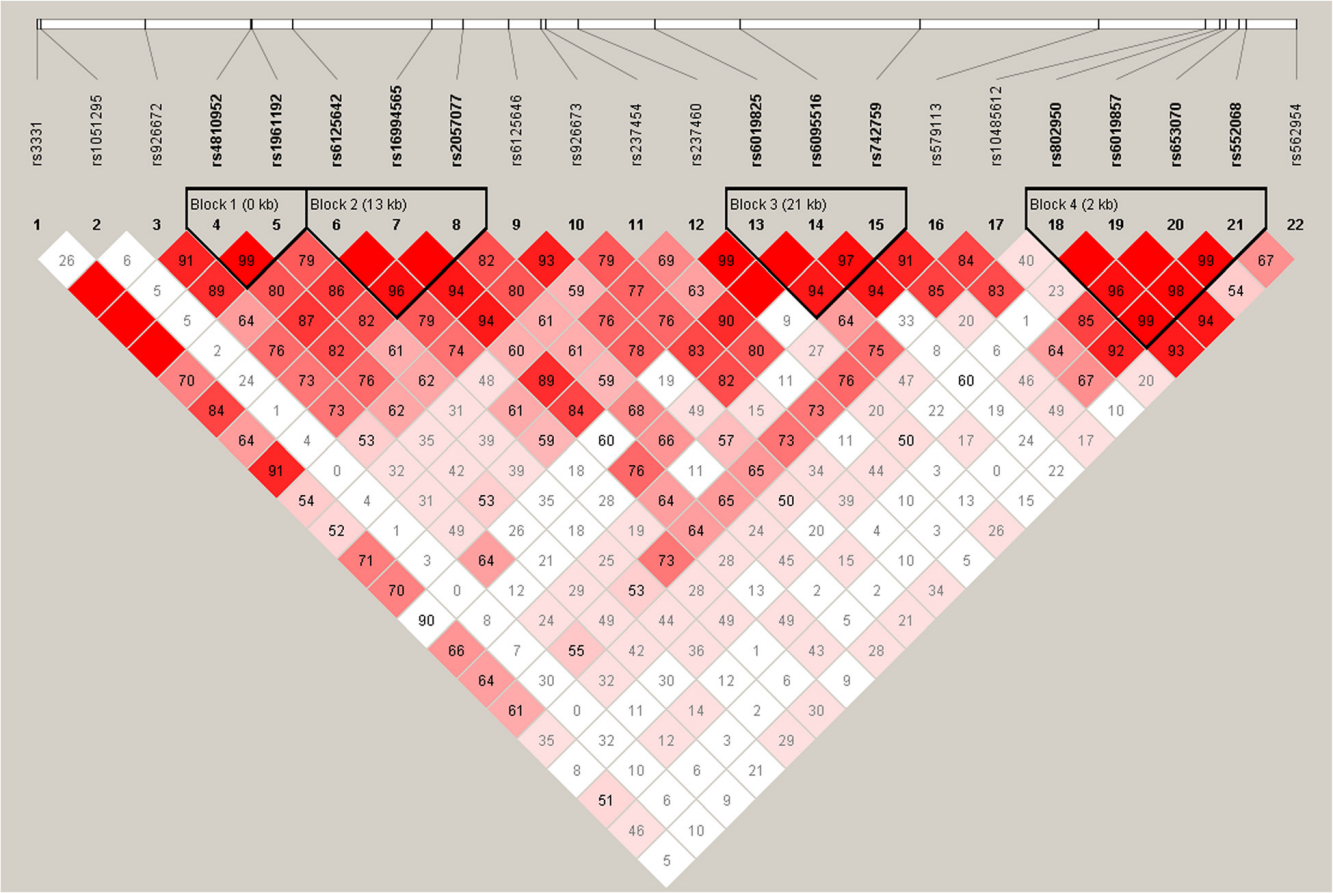
Pairwise LD pattern of the 22 tag SNPs in KCNB1 gene. Pairwise LD coefficients D′ × 100 are displayed in each cell

We constructed haplotypes for 22 tagging SNPs to examine whether these SNPs demonstrated any additional evidence of association with IR when tested together by using the Haploview program. We identified 4 haplotypes for the KCNB1 gene and each haplotype represented sites are as follows: Block1 (rs4810952-rs1961192),Block2 (rs6125642-rs16994546-rs2057077), Block3(rs6019852-rs6095516-rs742759), and Block4 (rs802950-rs6019857-rs653070-rs552068) (shown in Fig. 1). We found that the haplotype of Block 3 GCA was significantly different between the IR group and the control group (*P* = 0.04). Details are shown in Table 4. Rs926672 was not in a haplotype block generated in this study due to weak linkage disequilibrium with other SNPs.

**Table 4:** Association analysis of KCNB1 haplotypes with insulin resistance

Block	Frequency	Case, Control Ratio Counts	Case, Control Frequencies	*χ* ^2^	*P*
Block 1					
TT	0.47	183.0 : 203.0, 491.9 : 566.1	0.47, 0.47	0.09	0.76
CC	0.46	179.0 : 207.0, 484.9 : 573.1	0.46, 0.46	0.03	0.86
TC	0.07	24.0 : 362.0, 79.1 : 978.9	0.06, 0.08	0.66	0.42
Block 2					
TTC	0.48	186.8 : 199.2, 512.5 : 545.5	0.48, 0.48	0	0.99
GTT	0.38	145.8 : 240.2, 399.6 : 658.4	0.38, 0.38	0	0.99
TCC	0.08	26.0 : 360.0, 94.1 : 963.9	0.07, 0.09	1.73	0.20
Block 3					
ACG	0.48	182.6 : 203.4, 510.7 : 547.3	0.47, 0.48	0.10	0.75
GCG	0.36	152.3 : 233.7, 373.1 : 684.9	0.40, 0.35	2.15	0.14
GCA	0.09	23.7 : 362.3, 99.9 : 958.1	0.06, 0.09	3.95	0.04
Block 4					
CACA	0.52	193.7 : 192.3, 550.6 : 503.4	0.50, 0.52	0.48	0.49
AATC	0.18	69.9 : 316.1, 187.7 : 866.3	0.18, 0.18	0.02	0.90
CACC	0.13	56.0 : 330.0, 133.1 : 920.9	0.15, 0.13	0.89	0.35
CTTC	0.11	39.2 : 346.8, 124.8 : 929.2	0.10, 0.12	0.81	0.37

## Discussion

Here, we first report four genetically non-linked KCNB1 tag SNPs (rs237460, rs3331, rs16994565 and rs802950, all r^2^ < 0.65) to be associated with significant changes in serum TC levels, with two of these (rs237460 and rs802590) further associated or trend to be associated with reduced LDL-C levels. In addition, we also demonstrated that one novel tag SNPs rs926672 was associated with increased serum TG levels, in addition to the previously identified KCNB1 rs1051295 [2]. We also showed KCNB1 rs926672 associated with insulin resistance by a case–control study, and two tag SNPs (rs2057077and rs4810952) of KCNB1 were associated with the measure of insulin resistance as determined by HOMA-IR in a cross-section study. Together, these results indicate that KCNB1 is likely associated with changes in lipid metabolism and insulin sensitivity.

Although the role of Kv2.1 channel in lipid and fat metabolism has not been assessed, previous reports could provide some clues. Besides the simple diffusion manner, fatty acids, previously thought to be transported across the cell membrane by simple diffusion, is now known to also do so by competitive receptor-mediated transporters including fatty acid translocase (FAT), fatty acid binding protein (FABP) and fatty acid transport protein (FATP) [8–10]. Fatty acid transport protein-1 (FATP1) which transports free fatty acids (FFA) is insulin-sensitive and FATP1 expression in adipocytes is influenced by insulin-mediated transcriptional modulation [11]. Recent studies have shown that the interaction between the C-terminus of Kv2.1 channel and SNARE protein syntaxin-1A in islet beta cell is required for efficient insulin exocytosis and glucose-stimulated insulin secretion [6] . In addition, studies have demonstrated that FAT levels would be improved with adipocyte differentiation [12, 13], while Kv channels Kv2.1 and Kv3.3 were shown to possibly play a role in the differentiation of pre-adipocytes and human mesenchymal stem cells into adipocytes [14, 15]. These postulated mechanisms may underlie the association between SNPs of Kv2.1 channel and lipid metabolism, and a possible reason for these Kv2.1 SNPs being associated with insulin resistance, whereby dysregulated lipid metabolism is one of the risk factors for insulin resistance [16].

In addition, there was substantial evidence that internal interactions within some loci in gene might have a large impact on the phenotype we observed. Many studies have shown that neither single SNP loci nor multiple SNP loci have brought us greater effectiveness than haplotype-based research in the analysis of complex diseases [17]. Haplotype was likely to have more information about linkage disequilibrium, which made it easier to find and locate causative loci in association analysis [18, 19]. In this study we constructed four haplotypes for the KCNB1 gene and found Block 3 (rs6019852-rs6095516-rs742759) genotype GCA was significantly different distributed between the IR group and the control group (*P* = 0.04). This result indicated that the haplotype composed of three SNPs above is likely to have association with IR by internal interactions. In addition,we also found that KCNB1 rs926672 was not in any haplotype block generated in this study due to weak linkage disequilibrium with other SNPs, which suggests that rs926672 may be an independent genetic marker of insulin resistance.

KCNB1 SNPs have been showed to be associated with some diseases in human, such as rheumatoid arthritis, left ventricular hypertrophy, long QT syndrome and type 2 diabetes in previous studies [2, 20–22]. In this study, we demonstrated that eight of the 22 selected tag SNPs are associated with changes in serum lipids or insulin resistance, which adds new insight to our previous report showing the association of KCNB1 rs1051295 with type 2 diabetes [2]. Since these SNP are not located in the coding regions of KCNB1, we speculate that it might not influence channel pore kinetics per se, but rather it may influence the post-translational processing of the Kv2.1 channel. Further study should be aimed at elucidating at identifying the potential mechanisms that these SNPs exert their effects on lipid metabolism and insulin sensitivity.

The limitation of this study is the relative small sample size, thus, the association needs to be verified in a study with larger sample size within the Chinese Han population, and in other non-Chinese populations. Also, because the small sample size, we did not do a multiple testing correction for the significance threshold, although it was conservative for an explore study [23]. In view of lipid metabolism and insulin resistance are central to the etiology of many complex diseases, future research needs to focus on whether these KCNB1 SNPs are associated with these diseases as well.

## Conclusion

This study provides the initial evidence for the association between KCNB1 polymorphisms with lipid metabolism and insulin resistance in Chinese Han population. This initial study indicates that Kv2.1 may play a role in the lipid metabolism and thus influence insulin sensitivity. Further studies are needed to examine the precise mechanism underlying the association of KCNB1 polymorphisms with lipid metabolism and insulin resistance.

## Materials and methods

This study was approved by the Ethics Committee of Capital Medical University (Beijing, China) and was conducted in accordance with the principles of the Helsinki Declaration II. Written consent was obtained from all participants.

### Study participants and data collection

This cross-sectional study involved 733 Chinese Han participants included 315 males and 418 females without overt diabetes. Subjects were consecutively recruited at the Physical Examination Center, Beijing Xuanwu Hospital. Venous blood samples were drawn from all participants after an overnight fast of 8-10 h for DNA preparation and determination of fasting plasma glucose (FPG), fasting insulin (FINS), total cholesterol (TC), triglyceride (TG), high-density lipoprotein cholesterol (HDL-C) and low-density lipoprotein cholesterol (LDL-C). Height, weight, waist circumference (WC) and hip circumference (HC) of participants were measured according to standard protocols. Body mass index (BMI) was calculated by dividing the weight (in kg) by the height (in m) squared. Waist to hip ratio (WHR) was calculated by dividing the waist circumference by the hip circumference.

### Tag SNPs selection and genotyping

Genotyping data was obtained from the CHB (Han Chinese in Beijing) panel of the Build 36 of HapMap Project to choose the 22 tag SNPs within the range of the first and last exon (transcribed region) of KCNB1 on the basis of the following principal criteria: r^2^ > 0.8(100 % coverage) and minor allele frequency (MAF) > 0.05. Of those tag SNPs, 20 were located in introns, and 2 in the 3’-UTR (rs3331 and rs1051295). Characteristics of the selected tag SNPs, included genomic position, genic position, minor allele frequency, and Hardy-Weinberg equilibrium shown in Table 5.

**Table 5:** The information of KCNB1 tag SNPs

SNP ID	Genomic position (bp)	Genic position	Alleles (major/minor)	MAF	HWE (*P*)
rs10485612	49468115	intron	A/G	0.10	0.70
rs16994565	49404537	intron	T/C	0.06	0.33
rs1961192	49389770	intron	C/T	0.45	0.78
rs2057077	49407099	intron	C/T	0.32	0.97
rs6125646	49410872	intron	C/T	0.50	0.42
rs4810952	49389638	intron	C/T	0.39	0.61
rs6019825	49422880	intron	G/A	0.49	0.08
rs6019857	49469765	intron	A/T	0.10	0.83
rs6095516	49429848	intron	C/T	0.06	0.79
rs6125642	49393138	intron	G/T	0.39	0.58
rs1051295	49372368	3′-UTR	T/C	0.48	0.59
rs653070	49470871	intron	T/C	0.28	0.04
rs742759	49444608	intron	A/G	0.14	0.01
rs802950	49469295	intron	C/A	0.16	0.29
rs926673	49413473	intron	T/G	0.40	0.30
rs237454	49413854	intron	T/C	0.42	0.68
rs237460	49416590	intron	G/A	0.42	0.67
rs926672	49380954	intron	A/T	0.41	0.30
rs552068	49471461	intron	A/C	0.45	0.17
rs562954	49475539	intron	G/A	0.19	0.74
rs579113	49459375	intron	A/G	0.26	0.14
rs3331	49372064	3′-UTR	T/C	0.09	0.38

*MAF* minor allele frequency, *HWE* Hardy-Weinberg equilibrium

DNA was isolated from peripheral white blood cells using DNeasy tissue kit (Qiagen) and samples were diluted to a final concentration of 20-50 ng/ml. Genotyping was assessed using Mass Array (Sequenom, San Diego, CA) based on allele-specific MALDI-TOF mass spectrometry. DNA from samples was randomly assigned to 96 well plates, with genotyping performed in blinded manner.

PCR was performed for a 384-well microtiter plate of reactions in which the same assay was applied to different DNAs. Each PCR procedure was carried out in a total volume of 5 μL. The PCR cocktail contained 1 μL of genomic DNA, 1 μL of each primer, 0.5 μL of 10*PCR buffer, 0.1 μL deoxy-ribonucleoside triphosphate, 0.4 μL MgCl_2_, 0.2 μL Hotstar and 1.8 μL H_2_O. In the thermal reactor, amplification of the KCNB1 was achieved under the following conditions: denaturation at 94 °C for 4 min followed by 45 amplification cycles of (denaturation at 94 °C for 20 s, annealing at 56 °C for 30 s, extension at 72 °C for 1 min) and a final extension step of 3 min at 72 °C.

The genotyping success rates were > 98.5%and all tag SNPs are consistent with the Hardy-Weinberg equilibrium (*P* > 0.05) except for rs653070 and rs742759 for which the *P* value was 0.04 and 0.01, respectively, thus excluded from further analysis. Genotyping of all samples was repeated in 5 % of random samples for verification and quality control; all revealed the genotype data had an error rate of <1 %. The genetic linkage between tag SNPs ranged from ‘none’ (D’ = 0.00, r^2^ = 0.00) to ‘moderate’ (D’ = 1.00, r^2^ = 0.79, shown in Fig. 1).

### Definitions

The value of homeostasis model assessment (HOMA) denoting insulin resistance (IR) was termed as HOMA- IR and was calculated as equal to (fasting insulin [in IU/mL]*fasting glucose [in mmol/L]/22.5) [24]. Subjects having HOMA-IR values in the highest quartile (P_75_) were defined as insulin resistance in our study [25].

### Statistical analyses

Statistical analysis was performed using SPSS software, version 18.0 (SPSS, Inc, Chicago, IL. purchased by Capital Medical University, China). The quantitative variables of metabolic traits were expressed as mean ± SD. The difference of these variables among the 3 genotypes of tag SNPs or between IR and Non-IR groups was assessed by AVONA or independent *t* test, respectively. The *χ*^2^ test was used to examine the differences of SNPs or gender between the IR and Non-IR groups. The association between the tag SNPs and the metabolic traits or insulin resistance was finally determined by multiple linear regression analysis or unconditional logistic regression analysis, in which age and gender were adjusted as confounding factors, and *b* or *OR* and their 95 % confidence interval (CI) were presented.

The Haploview program (http://www.broad.mit.edu/mpg/haploview/) was used to select tag SNPs, calculate pairwise linkage disequilibrium statistics and examine allelic and haplotype associations with insulin resistance. The Haploview program was also used to draw the figure of pairwise LD pattern. Hardy-Weinberg equilibrium was determined for each SNP distribution. A value of *P* < 0.05 was considered as significant.

## Abbreviations

SNP: Single-nucleotide polymorphisms
TG: Triglycerides
TC: Total cholesterol
HDL-C: High-density lipoprotein cholesterol
LDL-C: Low-density lipoprotein cholesterol
FPG: Fasting plasma glucose
FINS: Fasting insulin
WHR: Waist to hip ratio
BMI: Body mass index
MAF: Minor allele frequency
HWE: Hardy-Weinberg equilibrium
CI: Confidence interval
IR: Insulin resistance

## References

[1] Gutman GA, Chandy KG, Grissmer S, Lazdunski M, McKinnon D, Pardo LA (2005). International Union of Pharmacology. LIII. Nomenclature and molecular relationships of voltage-gated potassium channels. Pharmacol Rev.

[2] Zhang Y-XX, Liu Y, Dong J, Wang Y-XX, Wang J, Zhuang G-QQ (2013). An exploratory study of the association between KCNB1 rs1051295 and type 2 diabetes and its related traits in Chinese Han population. PLoS ONE.

[3] Singer-Lahat D, Sheinin A, Chikvashvili D, Tsuk S, Greitzer D, Friedrich R (2007). K+ channel facilitation of exocytosis by dynamic interaction with syntaxin. J Neurosci.

[4] Feinshreiber L, Singer-Lahat D, Ashery U, Lotan I (2009). Voltage-gated potassium channel as a facilitator of exocytosis. Ann N Y Acad Sci.

[5] Feinshreiber L, Singer-Lahat D, Friedrich R, Matti U, Sheinin A, Yizhar O (2010). Non-conducting function of the Kv2.1 channel enables it to recruit vesicles for release in neuroendocrine and nerve cells. J Cell Sci.

[6] Dai XQ, Manning Fox JE, Chikvashvili D, Casimir M, Plummer G, Hajmrle C (2012). The voltage-dependent potassium channel subunit Kv2.1 regulates insulin secretion from rodent and human islets independently of its electrical function. Diabetologia.

[7] Upadhyay SK, Eckel-Mahan KL, Mirbolooki MR, Tjong I, Griffey SM, Schmunk G (2013). Selective Kv1.3 channel blocker as therapeutic for obesity and insulin resistance. Proc Natl Acad Sci U S A.

[8] Lobo S, Wiczer BM, Smith AJ, Hall AM, Bernlohr DA (2007). Fatty acid metabolism in adipocytes: functional analysis of fatty acid transport proteins 1 and 4. J Lipid Res.

[9] Doege H, Stahl A (2006). Protein-mediated fatty acid uptake: novel insights from in vivo models. Physiology (Bethesda).

[10] Nickerson JG, Alkhateeb H, Benton CR, Lally J, Nickerson J, Han X-XX (2009). Greater transport efficiencies of the membrane fatty acid transporters FAT/CD36 and FATP4 compared with FABPpm and FATP1 and differential effects on fatty acid esterification and oxidation in rat skeletal muscle. J Biol Chem.

[11] Wu Q, Ortegon AM, Tsang B, Doege H, Feingold KR, Stahl A (2006). FATP1 is an insulin-sensitive fatty acid transporter involved in diet-induced obesity. Mol Cell Biol.

[12] Hajri T, Abumrad NA (2002). Fatty acid transport across membranes: relevance to nutrition and metabolic pathology. Annu Rev Nutr.

[13] Laugerette F, Passilly-Degrace P, Patris B, Niot I, Febbraio M, Montmayeur J-PP (2005). CD36 involvement in orosensory detection of dietary lipids, spontaneous fat preference, and digestive secretions. J Clin Invest.

[14] You MH, Song MS, Lee SK, Ryu PD, Lee SY, Kim DY (2013). Voltage-gated K+ channels in adipogenic differentiation of bone marrow-derived human mesenchymal stem cells. Acta Pharmacol Sin.

[15] Ramírez-Ponce MP, Mateos JC, Bellido JA (2002). Insulin increases the density of potassium channels in white adipocytes: possible role in adipogenesis. J Endocrinol.

[16] Liu J, Jahn LA, Fowler DE, Barrett EJ, Cao W, Liu Z (2011). Free fatty acids induce insulin resistance in both cardiac and skeletal muscle microvasculature in humans. J Clin Endocrinol Metab.

[17] Chen X, Li Z (2008). Inference of haplotype effects in case-control studies using unphased genotype and environmental data. Biom J.

[18] Clark AG (2004). The role of haplotypes in candidate gene studies. Genet Epidemiol.

[19] Morris RW, Kaplan NL (2002). On the advantage of haplotype analysis in the presence of multiple disease susceptibility alleles. Genet Epidemiol.

[20] Xiao X, Zhang Y, Wang K (2009). Association of KCNB1 to rheumatoid arthritis via interaction with HLA-DRB1. BMC Proc.

[21] Arnett DK, Li N, Tang W, Rao DC, Devereux RB, Claas SA (2009). Genome-wide association study identifies single-nucleotide polymorphism in KCNB1 associated with left ventricular mass in humans: the HyperGEN Study. BMC Med Genet.

[22] Iwasa H, Kurabayashi M, Nagai R, Nakamura Y, Tanaka T (2001). Multiple single-nucleotide polymorphisms (SNPs) in the Japanese population in six candidate genes for long QT syndrome. J Hum Genet.

[23] William Stafford N (2009). How does multiple testing correction work. Nat Biotechnol.

[24] Resnick HE, Jones K, Ruotolo G, Jain AK, Henderson J, Lu W (2003). Insulin resistance, the metabolic syndrome, and risk of incident cardiovascular disease in nondiabetic american indians: the Strong Heart Study. Diabetes Care.

[25] Hanson RL, Imperatore G, Bennett PH, Knowler WC (2002). Components of the “metabolic syndrome” and incidence of type 2 diabetes. Diabetes.

