# Erratum to: Association of KCNB1 polymorphisms with lipid metabolisms and insulin resistance: a case-control design of population-based cross-sectional study in Chinese Han population

**DOI:** 10.1186/s12944-015-0144-9

**Published:** 2015-11-06

**Authors:** Yuncui Yu, Jing Wang, Ruiying Kang, Jing Dong, Yuxiang Zhang, Fen Liu, Yuxiang Yan, Rong Zhu, Lili Xia, Xiaoxia Peng, Ling Zhang, Dian He, Herbert Y. Gaisano, Zhenwen Chen, Yan He

**Affiliations:** School of Public Health, Capital Medical University, No.10 Xitoutiao, Youanmen, Fengtai District, Beijing, 100069 PR China; Departments of Emergency, Beijing Xuanwu Hospital, Capital Medical University, No.45Changchun Street, Xuanwu District, Beijing, Beijing 100053 PR China; Departments of Medicine and Physiology, University of Toronto, 315 Bloor Street West, Toronto, Ontario Canada; School of Basic Medicine, Capital Medical University, No.10 Xitoutiao, Youanmen, Fengtai District, Beijing, 100069 PR China

## Erratum

Unfortunately, the original version of this article [1] contained an error in the author list.

Herbert Y. Gaisano was incorrectly listed as ‘Gaisano Herbert’. The original article has now been updated to reflect the correct author name.
